# Earthquake Forecasting Based on *b* Value and Background Seismicity Rate in Yunnan Province, China

**DOI:** 10.3390/e27020205

**Published:** 2025-02-15

**Authors:** Yuchen Zhang, Rui Wang, Haixia Shi, Miao Miao, Jiancang Zhuang, Ying Chang, Changsheng Jiang, Lingyuan Meng, Danning Li, Lifang Liu, Youjin Su, Zhenguo Zhang, Peng Han

**Affiliations:** 1 Department of Astronautical Science and Mechanics, Harbin Institute of Technology, Harbin 150001, China; 11849527@mail.sustech.edu.cn; 2 Department of Earth and Space Sciences, Southern University of Science and Technology, Shenzhen 518055, China; wangr9@sustech.edu.cn (R.W.); miaom@sustech.edu.cn (M.M.); zhangzg@sustech.edu.cn (Z.Z.); 3 Key Laboratory of Earthquake Forecasting and Risk Assessment, Ministry of Emergency Management, Southern University of Science and Technology, Shenzhen 518055, China; 4 China Earthquake Networks Center, Beijing 100045, China; shihaixia08@seis.ac.cn (H.S.); menglingyuan@seis.ac.cn (L.M.); 5 Institute of Statistical Mathematics, Tokyo 190-8562, Japan; zhuangjc@ism.ac.jp; 6 BGRIMM Technology Group, Institute of Mining Engineering, Beijing 100160, China; changy@bgrimm.com; 7 Institute of Geophysics, China Earthquake Administration, Beijing 100081, China; jiangcs@cea-igp.ac.cn; 8 Earthquake Administration of Yunnan Province, Kunming 650224, China; zuni_2001@163.com (D.L.); lifang_l@sina.com (L.L.); suyoujin0818@sina.com (Y.S.)

**Keywords:** earthquake forecast, *b* value, background seismicity rate, Molchan Error Diagram, Yunnan Province

## Abstract

Characterized by frequent earthquakes and a dense population, Yunnan Province, China, faces significant seismic hazards and is a hot place for earthquake forecasting research. In a previous study, we evaluated the performance of the *b* value for 5-year seismic forecasting during 2000–2019 and made a forward prediction of M ≥ 5.0 earthquakes in 2020–2024. In this study, with the forecast period having passed, we first revisit the results and assess the forward prediction performance. Then, the background seismicity rate, which may also offer valuable long-term forecasting information, is incorporated into earthquake prediction for Yunnan Province. To assess the effectiveness of the prediction, the Molchan Error Diagram (MED), Probability Gain (PG), and Probability Difference (PD) are employed. Using a 25-year catalog, the spatial *b* value and background seismicity rate across five temporal windows are calculated, and 86 M ≥ 5.0 earthquakes as prediction samples are examined. The predictive performance of the background seismicity rate and *b* value is comprehensively tested and shown to be useful for 5-year forecasting in Yunnan. The performance of the *b* value exhibits a positive correlation with the predicted earthquake magnitude. The synergistic effect of combining these two predictors is also revealed. Finally, using the threshold corresponding to the maximum *PD*, we integrate the forecast information of background seismicity rates and the *b* value. A forward prediction is derived for the period from January 2025 to December 2029. This study can be helpful for disaster preparedness and risk management in Yunnan Province, China.

## 1. Introduction

The Gutenberg–Richter law (G-R law) describes the relationship between earthquake magnitude (M) and the number of earthquakes (*N*) using the formula ${log}_{10}\left(N\right)=a-bM$, where ‘a’ and ‘b’ represent constants for the seismicity and the slope, respectively [1,2]. Several studies have identified the presence of a low *b* value in the vicinity of the epicenter prior to major seismic events. Notable examples include the 2004 M 9.0 Sumatra earthquake, the 2008 M 8.0 Wenchuan earthquake, and the 2011 M 9.0 Tohoku earthquake [3,4,5,6,7]. These observations suggest a potential relationship between a low *b* value and the occurrence of significant seismic activity [8,9,10]. Some studies on natural time analysis of earthquake catalogs emphasize the pivotal role of entropy in assessing earthquake risk and reveal that a decrease in the *b* value prior to large earthquakes corresponds to an increase in order parameter fluctuations as the system approaches the critical point (i.e., the mainshock) [11,12,13]. Further analysis from laboratory experiments demonstrates a consistent decrease in *b* value with increasing stress levels [14]. This finding establishes a clear relationship between the *b* value and stress differentials, providing robust evidence to support an earthquake prediction methodology that utilizes the *b* value as a key indicator [15,16,17,18,19,20,21].

To quantitatively assess seismic risk using the *b* value, Wang et al. (2021) partitioned earthquake data from January 2000 to December 2019 into four distinct five-year periods and evaluated forecasting efficiency using the Molchan Error Diagram (MED), Probability Gain (PG), and Probability Difference (PD) [22,23] in Yunnan Province, China. They then utilized the spatial *b* value from January 2015 to December 2019 to identify high-earthquake-potential regions in Yunnan. As of 2025, the predicted time window has elapsed, allowing a rigorous assessment of the forward prediction performance. On the other hand, the *b* value has a larger error and is less informative in the regions with very low seismicity. A promising direction for improvement is the incorporation of additional earthquake prediction information, such as background seismicity rate, into the existing framework.

Previous studies suggest that background seismicity rate is a valuable factor for earthquake prediction. According to the rate–state model (RSM), there is a positive correlation between seismicity rate and stress changes, with regional stress loading playing a dominant role in background seismicity rates [24,25,26,27,28,29,30]. Numerous studies have established relationships among background seismic rates, strain rates, and underground fluid signals [25,31,32]. And numerical simulations have demonstrated that seismic activity varies with regional stress levels [21,33]. Statistical studies indicate that major earthquakes are more likely to occur in regions with high background seismicity rates [34,35]. The background seismicity rate is a critical reference for middle–long-term earthquake forecasts and is commonly employed to assess earthquake hazards [34,35,36,37].

This study aims to identify high-earthquake-potential regions during 2025–2029 for disaster preparedness and risk management in Yunnan Province, China. First, we revisit the previous 5-year forward prediction for 2020–2024. Then, the forecast performance of the spatial *b* value and background seismicity rate are examined using the data for 2000–2024, and the synergistic effect of combining these two predictors is investigated. Finally, based on the test results, a strategy for integrating the forecast information of background seismicity rates and the *b* value is proposed, and a forward prediction is derived for 2025–2029.

## 2. Data

The earthquake catalog used in this study to estimate the *b* value and background seismicity rate is obtained from the China Earthquake Networks Center (CENC, http://www.ceic.ac.cn). Following the methodology outlined by Wang et al. (2021), our study area is defined by the coordinates 97° E–106.5° E and 21° N–30° N. A magnitude threshold of 3.0 was utilized in accordance with the previous study to ensure the completeness of the earthquake data [23,38].

## 3. Methods

### 3.1. b Value Estimation

By the Gutenberg–Richter law (G-R law), the probability density function governing earthquake magnitude can be formally expressed as

(1) $$f\left(M\right)=\frac{N(M)}{\int_{M_{c}}^{\infty }N\left(M\right)dM}=\beta e^{-\beta (M-M_{c})},$$

The likelihood function for a specific set of earthquake samples with magnitudes $M_{1}$, $M_{2}$,……, $M_{n}$ is defined as

(2) $$L\left(\beta \right)=\prod_{i=1}^{n}f_{\beta }\left(M_{i}\right)=\prod_{i=1}^{n}\beta e^{-\beta (M_{i}-M_{c})},$$

In this context, $M_{c}$ represents the complete magnitude. Ogata extends the *b* value to be contingent upon location and postulates that β is a function of the epicenter coordinates $\left(x_{i},y_{i}\right)$, denoted as $\beta =\beta (x_{i},y_{i})$ [34,39]. Given the inherently positive nature of the *b* value, $\beta (x_{i},y_{i})$ can be parameterized as

(3) $$\beta \left(x,y\right)=e^{\phi_{\theta }(x,y)},$$

Here, $\phi_{\theta }$ denotes the 2-D B-spline function, while *θ* represents the coefficients associated with $\phi_{\theta }$. Consequently, β can be expressed as a flexible function dependent on spatial coordinates.

In the Hierarchical Space–Time Point-Process Models (HIST-PPM), the study area is partitioned into Delaunay triangles centered on earthquake epicenters. To mitigate the risk of overfitting, Ogata estimated the parameter *θ* by maximizing the penalized log-likelihood function, formulated as follows [39,40]:

(4) $$R\left(\theta |w\right)=lnL\left(\theta \right)-Q(\theta |w),$$

Here, $Q\left(\theta |w\right)$ represents the penalty term, defined as

(5) $$Q\left(\theta |w\right)=w\iint \left\{{\left(\frac{\partial \phi_{\theta }(x,y)}{\partial x}\right)}^{2}+{\left(\frac{\partial \phi_{\theta }(x,y)}{\partial y}\right)}^{2}\right\}dxdy,$$

The weight, denoted as w, is a hyperparameter that can be optimized by the Akaike Bayesian Information Criterion (ABIC) [41,42]. The ABIC is expressed as

(6) $$ABIC=-2\max\left(logL\right)+2(number\;of\;\;hyperparameters),$$

By minimizing the ABIC, the optimal set of hyperparameters can be estimated, thereby yielding the best fit to the data.

The computation of the *b* value at the epicenter is attainable using the optimized parameters *θ*, as outlined in Equation (3). Furthermore, the *b* value within the triangular mesh can be estimated through linear interpolation. This process results in a spatial distribution of *b* values across the entirety of Yunnan Province, with a resolution of 0.1° × 0.1°.

### 3.2. Background Seismicity Rate

Zhuang et al. (2002) proposed an iterative algorithm that can estimate background seismicity rate based on a space–time Epidemic-Type Aftershock Sequence (ETAS) [43]. The conditional intensity functions of spatial–temporal sequences with i earthquakes $\left(t_{i},x_{i},y_{i},M_{i}\right)$ are formulated as follows:

(7) $$\lambda (t,x,y)=\mu \left(x,y\right)+\sum_{i;t_{i}<t}\kappa (M_{i})g(t-t_{i})f(x-x_{i},y-y_{i},M_{i}),$$

Here, λ(t,x,y) represents the seismicity rate, indicating the number of earthquakes per unit time and space at time t and coordinates (x,y). The first item on the right side of Equation (7), $\mu \left(x,y\right)$, denotes the spatial background seismicity rate. The term $\kappa (M_{i})g(t-t_{i})f(x-x_{i},y-y_{i},M_{i})$ is the contribution of the *i*-th earthquake occurring before time *t*. And $\kappa \left(M\right)$ is the mean number of earthquakes directly triggered by an earthquake of magnitude M, expressed as

(8) $$\kappa \left(M\right)=Ae^{\alpha (M-M_{c})},\;\;M\geq M_{c},$$

The probability density function for the occurrence times and locations of directly triggered events is given by

(9) $$g\left(t\right)=\frac{p-1}{c}{\left(1+\frac{t}{c}\right)}^{-p},\;\;t>0,$$

(10) $$f\left(x,y;M\right)=\frac{q-1}{\pi De^{\gamma \left(M-M_{c}\right)}}{\left(1+\frac{x^{2}+y^{2}}{De^{\gamma \left(M-M_{c}\right)}}\right)}^{-q},$$

The parameters $\left(A,\alpha ,c,p,D,q,\gamma \right)$ can be estimated by maximizing the likelihood function:

(11) $$lnL=\sum_{i:\left(t_{i},x_{i},y_{i}\right)\in S\times [0,T]}ln\lambda \left(t_{i},x_{i},y_{i}\right)-\int_{0}^{T}\iint_{S}\lambda (t,x,y)dxdydt$$

Using these parameters, the probability that the i-th earthquake is a background event can be estimated as

(12) $$\varphi_{i}=\frac{\mu \left(x_{i},y_{i}\right)}{\lambda (t_{i},x_{i},y_{i})}$$

With $\varphi_{i}$ for all the earthquakes obtained, the $\mu \left(x,y\right)$ can be estimated by smoothing the background. Hence, a variable kernel is employed:

(13) $$\hat{\mu }\left(x,y\right)=\frac{1}{T}\sum_{i}\varphi_{i}Z_{d_{i}}(x-x_{i},y-y_{i})$$

Here, $Z_{d}$ denotes the Gaussian kernel function with bandwidth d, which is calculated as follows: For a given integer $n_{p}$, a circle is centered at the i-th earthquake with a radius $d_{i}$ that exceeds a minimum value ε. This circle must encompass at least $n_{p}$ earthquakes. In this study, $n_{p}$ and ε are set to 3 and 0.02, respectively, referring to [37]. By taking the smoothing background in Equation (13) as the background seismicity rate in Equation (7) and then estimating the parameters $\left(A,\alpha ,c,p,D,q,\gamma \right)$ through maximization as outlined in Equation (11), one can iteratively refine the estimates. This process allows for the determination of the background seismicity rate through multiple iterations. For further details, please refer specifically to [43].

### 3.3. Evaluation of Forecast Efficiency

The seismic forecast efficiency of the prior investigation is assessed using the MED [22,44,45]. This methodology utilizes a range of values, denoted as *thr*, which serves as a threshold spanning from the minimum to the maximum values observed. In relation to the *b* value, grid cells with *b* values lower than *thr* are considered alarmed. For the background seismicity rate, grid cells with rates higher than *thr* are alarmed. The ratio of alarmed grid cells is referred to as the alarming rate *τ*. An earthquake is considered detected if it occurs within the alarmed grid cells. The ratio of detected earthquakes is referred to as the detecting rate *ν*, while the rate of missed earthquakes is expressed as the missing rate 1 − *ν*. The curve in the diagram illustrates the relationship between the alarming and missing rates. In the case of a random forecast, the alarming rate is approximately equal to the detecting rate, resulting in a curve that aligns with the diagonal. When the forecasting efficiency exceeds that of a random guess, the alarming rate is lower than the detecting rate, resulting in a curve that falls below the diagonal. Conversely, if the alarming rate exceeds the detecting rate, the curve rises above the diagonal, indicating a less effective forecast than a random guess.

To further quantify forecasting efficiency, the *PG*, defined as the ratio of the detecting rate to the alarming rate, is expressed as follows [46,47]:

(14) $$PG=\frac{\nu }{\tau }$$

*PG* = 1 indicates that the forecasting efficiency is comparable to that of a random guess. A *PG* value greater than 1 signifies that the prediction outperforms a random guess, with higher *PG* values reflecting superior predictive performance.

Another metric employed to quantify forecasting performance in this study is the *PD*, defined as the difference between the gain (detecting rate) and the cost (alarming rate) [47].

(15) PD=ν−τ

In the context of a random prediction, the estimated *PD* is 0. A *PD* greater than 0 indicates that the prediction strategy outperforms a random forecast. Similar to the *PG*, a higher *PD* reflects superior predictive performance.

## 4. Results

### 4.1. Revisit of Earthquake Prediction in 2020–2024

**Figure 1:**
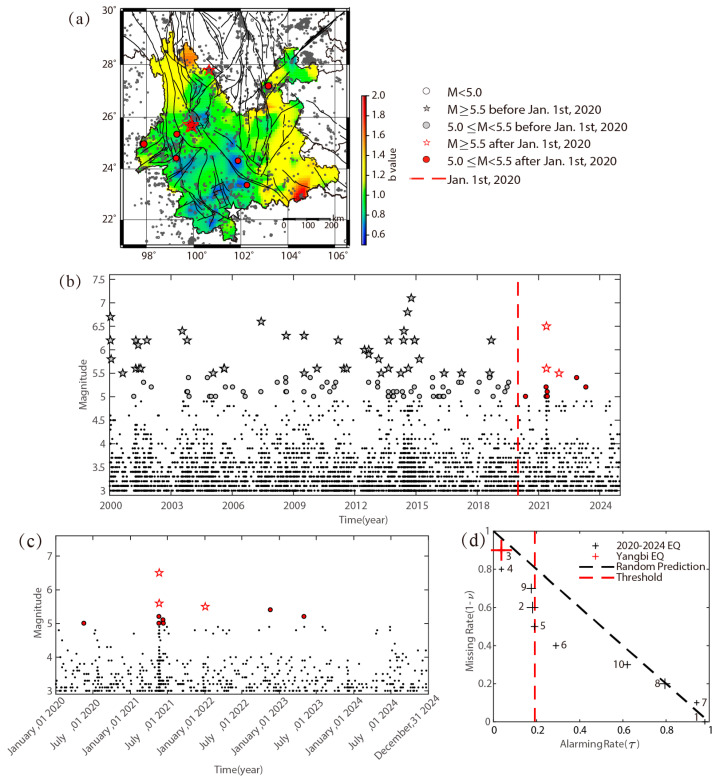
(**a**) The *b* value from January 2015 to December 2019 and earthquakes with M ≥ 5.0 from January 2020 to December 2024. The dot and star are scaled to the magnitude. (**b**) Temporal distribution of earthquakes in Yunnan Province from January 2000 to December 2024. (**c**) Temporal distribution of earthquakes in Yunnan Province from January 2020 to December 2024. (**d**) The MED of forecast performance based on the *b* value in (**a**). The marked numbers are the serial numbers in Table 1, and the size of the cross markers is scaled to the magnitude.

In the study by Wang et al. (2021), the identification of regions with high earthquake potential for the period from January 2020 to December 2024 is based on the *b* value observed between January 2015 and December 2019 [23]. Table 1 details the 10 earthquakes with M ≥ 5.0 and 3 earthquakes with M ≥ 5.5 that occurred in Yunnan Province during the forecast period, with their locations illustrated in Figure 1a, including the significant 2021 M6.5 Yangbi earthquake. Notably, on 24 December 2021, an earthquake of magnitude M6.0 occurred near the southern target area (see Figure 8 in the study of Wang et al., 2021 [23]) in Laos. Figure 1b,c display the magnitude–time diagram for earthquakes occurring in 2000–2024 and 2020–2024, respectively.

As detailed in Figure 1d, from January 2020 to December 2024, the observed data show that 2/3 of earthquakes with M ≥ 5.5 and 5/10 of those with M ≥ 5.0 occurred to the left of the threshold proposed in Wang et al. (2021) [23]. Notably, the 2021 M6.5 Yangbi earthquake (Serial Number 3) occurred at the place with the lowest *b* value among the ten events and could be predicted at the alarming rate of less than 5%. Although most of the earthquakes are under the diagonal line indicating clear forecast efficiency, the prediction is still far from practical demand. As the *b* value is hard to estimate and may become less useful if the seismicity is too low, the incorporation of additional earthquake prediction information, such as background seismicity rate, into the existing framework may have to be made.

### 4.2. b Value and Background Seismicity Rate in Yunan Province

**Figure 2:**
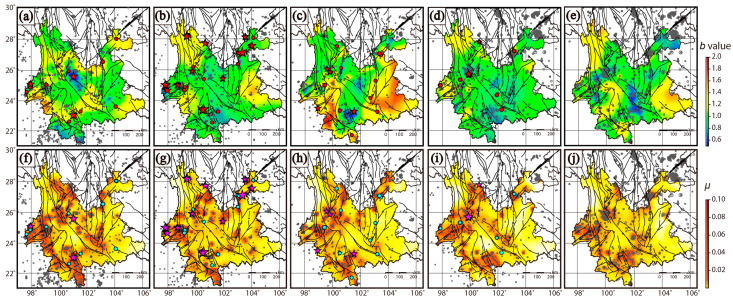
The *b* value and background seismicity rate. (**a**–**e**) *b* value; (**f**,**j**) background seismicity rate. Results in (**a**,**f**) using catalog in 2000–2004 and forecasting moderate–large earthquakes in 2005–2009; (**b**,**g**) using catalog in 2005–2009 and forecasting moderate–large earthquakes in 2010–2014; (**c**,**h**) using catalog in 2010–2014 and forecasting moderate–large earthquakes in 2015–2019; (**d**,**i**) using catalog in 2015–2019 and forecasting moderate–large earthquakes in 2020–2024; (**e**,**j**) using catalog in 2020–2024. A dot represents an earthquake with 5.0 ≤ M < 5.5. A star represents an earthquake with M ≥ 5.5. The sizes of the dots and stars are scaled to magnitude.

Figure 2 illustrates the *b* values and background seismicity rates across five periods, along with the earthquakes in the subsequent period. From January 2000 to December 2024, the *b* value exhibits significant variability, and its earthquake forecasting performance has been evaluated in prior studies, as noted above. The spatial distribution of background seismicity across the five periods reveals consistent patterns. Notably, there appears to be no discernible correlation between the *b* value and the background seismicity rate. Background seismic activity is notably pronounced in the northwest, west, southwest, and south regions of Yunnan Province, corresponding with the occurrence of moderate to large earthquakes in these areas. In contrast, background seismicity is comparatively low in the central and eastern parts of Yunnan, indicating a spatial variation in seismic activity throughout the province.

### 4.3. Comprehensive Forecast Performance

The time–space alarm model is employed to comprehensively evaluate the performance of the spatial *b* value and background seismicity rates. This model encompasses five distinct periods: January 2000 to December 2004, January 2005 to December 2009, January 2010 to December 2014, January 2015 to December 2019, and January 2020 to December 2024. During the interval from January 2005 to December 2024, the anticipated moderate to large earthquakes include 29 events with M ≥ 5.5 and 86 events with M ≥ 5.0.

**Figure 3:**
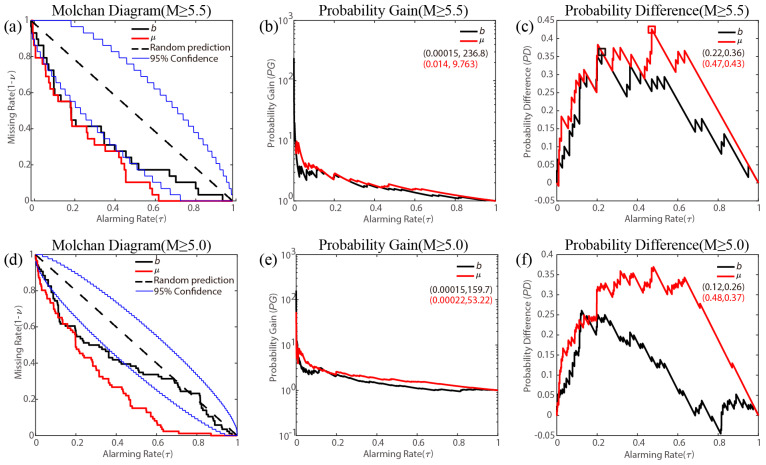
Forecast performance based on *b* value and background seismicity rate during 2005–2024. (**a**–**c**) show the results of earthquakes with M ≥ 5.5. (**a**) MED; (**b**) *PG*; (**c**) *PD*. (**d**–**f**) are the results of earthquakes with M ≥ 5.0. (**d**) MED; (**e**) *PG*; (**f**) *PD*. The number of earthquake samples is =29 and $N_{M\geq 5.0}=86$.

The forecasting performance of *b* values and background seismicity rates from January 2005 to December 2024 is evaluated and tested, as illustrated in Figure 3. In Figure 3a, the forecast efficiency for earthquakes with M ≥ 5.5 using both the *b* value and background seismicity rate approaches and partially exceeds the 95% significance level. This indicates that both predictors perform better than a random guess. Additionally, as shown in Figure 3d, the forecast efficiency for earthquakes with M ≥ 5.0 based on the *b* value significantly exceeds that of random predictions, with an alarming rate of less than 0.4. Furthermore, the forecasting efficiency of the background seismicity rate is significantly better than a random guess and notably higher than that of the *b* value.

The *PD* for the *b* value and background seismicity rate, as depicted in Figure 3b,e for earthquakes with M ≥ 5.5 and M ≥ 5.0, consistently exceeds 1. Specifically, the highest *PG* values for M ≥ 5.5 are 236.8 and 9.763 for the *b* value and background seismicity rate, respectively, while for M ≥ 5.0, they are 159.7 and 53.22. All of them are observed at very low alarming rates (below 0.01). In Figure 3c, the *PD* for earthquakes of magnitude M ≥ 5.5 remains consistently above 0. The highest *PD* values for M ≥ 5.5 are 0.36 for the *b* value and 0.43 for the background seismicity rate, corresponding to alarming rates of 0.22 and 0.47, respectively. For M ≥ 5.0, the highest *PD* values are 0.26 and 0.37, with alarming rates of 0.12 and 0.48. In summary, the *b* value demonstrates a higher *PG*, while the background seismicity rate shows a superior *PD*. The above evaluation of forecasting efficiency indicates that both the *b* value and background seismicity rate are effective predictors for middle–long-term earthquake occurrences.

**Figure 4:**
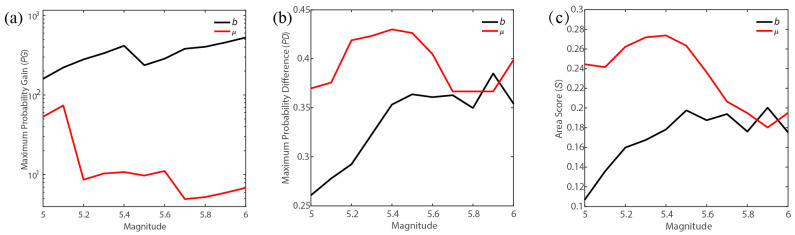
The variation in forecast performance with earthquake magnitude. (**a**) Variation in maximum *PG* with the forecast magnitude; (**b**) variation in maximum *PD* with the forecast magnitude; (**c**) variation in *S* with the forecast magnitude.

In Figure 3, the *PG* and *PD* for the *b* value appear to increase with the forecasted magnitude, whereas no such trend is observed for the background seismicity rate. This discrepancy likely reflects the differing efficiency of these two factors in earthquake forecasting across varying magnitudes. For further testing, we analyzed the variation in maximum *PG* and *PD* for both the *b* value and background seismicity rate with magnitude, as shown in Figure 4a,b. To quantitatively compare the effectiveness of earthquake forecasts, we employed the modified area score (*S*), which represents the area between the actual prediction curve and the random prediction diagonal line, as depicted in Figure 4c [47]. The maximum *PG*, *PD*, and *S* for the *b* value clearly increase with magnitude. While for the background seismicity rate, the maximum *PG*, *PD*, and *S* have no obvious variation trend. These results suggest that the *b* value might be more sensitive to and informative about large earthquakes in the study region.

**Figure 5:**
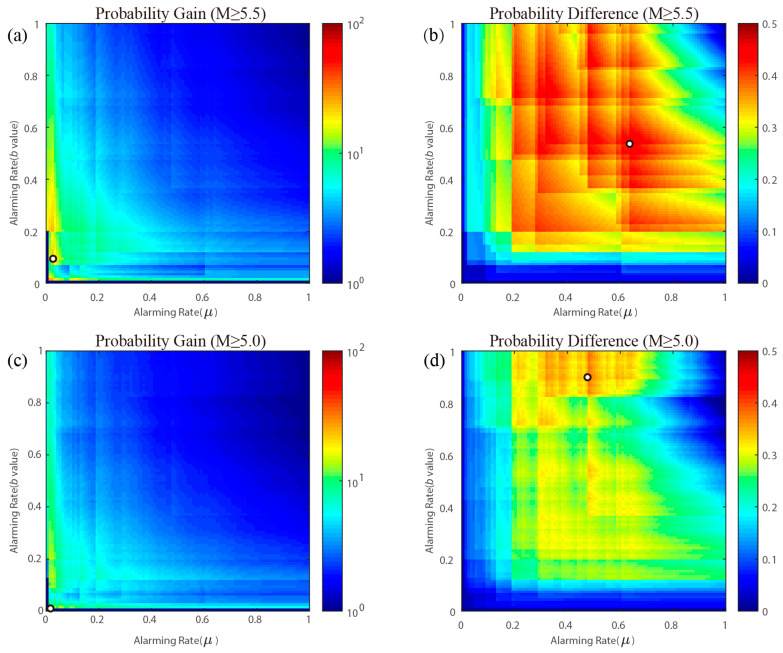
Forecast performance by combining *b* value and background seismicity rate during 2005–2024. The x-axis is the alarming rate of background seismicity corresponding to ${thr}_{\mu }$, and the y-axis is the alarming rate of the *b* value corresponding to ${thr}_{b}$. (**a**) *PG* for M ≥ 5.5 earthquakes; (**b**) *PD* for M ≥ 5.5 earthquakes; (**c**) *PG* for M ≥ 5.0 earthquakes; (**d**) *PD* for M ≥ 5.0 earthquakes. The location of the maximum value (*PG* or *PD*) in each figure is marked with dots and detailed in Table 2. The cross in (**b**) is located at the alarming rate corresponding to the maximum *PD* in Figure 3c.

To integrate prediction information from both the background seismicity rate and the *b* value, we conduct a comprehensive evaluation. We establish two thresholds: one for the *b* value (denoted as ${thr}_{b}$) and another for the background seismicity rate (denoted as ${thr}_{\mu }$). Grid cells with a *b* value below the threshold ${thr}_{b}$ and background seismicity rate above ${thr}_{\mu }$ would be alarmed. These two thresholds change gradually, and as a result, the alarming rates change correspondingly in the range 0–1.0. The overall forecast performance, which integrates both the *b* value and the background seismicity rate, is illustrated in Figure 5. Table 2 presents the maximum values and their respective locations, along with comprehensive alarming and detecting rates.

Effective seismic forecasting must balance both low alarming rates and high detecting rates. As illustrated in Table 2, for maximum *PG*, the alarming rates for earthquakes of M ≥ 5.5 and M ≥ 5.0 are 0.0052 and 0.0014, respectively, while the corresponding detecting rates are 0.14 and 0.023. The detecting rates, which fall below 0.2, suggest that using this threshold for reliable earthquake forecasting may result in a high missing rate. Conversely, for maximum *PD*, the alarming rates for M ≥ 5.5 and M ≥ 5.0 are 0.36 and 0.43, with detecting rates of 0.83 and 0.81, respectively. Yet the alarming rates exceed 0.3 and may cause large false alarms. Both the maximum *PD* and maximum *PG* fail to establish a suitable threshold for effective earthquake prediction, indicating that further exploration is needed to determine an applicable threshold.

## 5. Implication and Application

**Figure 6:**
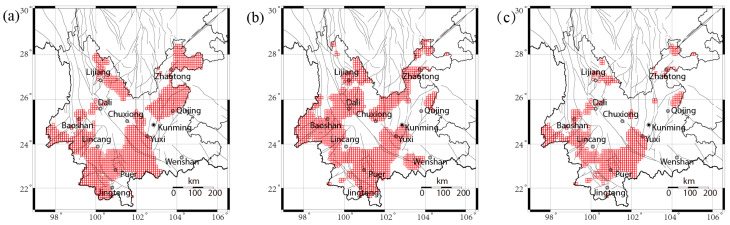
Alarmed regions for the period from January 2025 to December 2029 based on *b* value and background seismicity rate obtained during 2020–2024. (**a**) Alarmed area based on *b* value and ${thr\_PD}_{b}$, with 0.38 alarming rate; (**b**) alarmed area based on background seismicity rate and ${thr\_PD}_{\mu }$, with 0.42 alarming rate; (**c**) alarmed area based on *b* value and background seismicity rate, with 0.20 alarming rate. The red edge squares show the alarmed grid cells.

Taking a step back, the thresholds used for forecasting are determined based on the *PD* of the *b* value and background seismicity rate. Using the maximum *PD* for M ≥ 5.5 in Figure 3c and data from 2000 to 2024, the *b* value and background seismicity rate corresponding to the maximum *PD* are set as the thresholds, denoted as ${thr\_PD}_{b}$ and ${thr\_PD}_{\mu }$, respectively, and summarized in Table 3. If earthquake forecasting relies solely on either the *b* value or the background seismicity rate, the *PG* is 2.64 or 1.91, respectively. However, when integrating the *b* value and background seismicity rate, the *PG* increases to 3.69. This difference in *PG* suggests that incorporating the background seismicity rate contributes to an improvement in forecasting performance. The earthquake forecast for the period from January 2025 to December 2029 is based on the spatial *b* value and background seismicity rate data from January 2020 to December 2024, as illustrated in Figure 2e,j. Based on the *b* value, grid cells with a *b* value lower than ${thr\_PD}_{b}$ are alarmed, as shown in red in Figure 6a. Based on the background seismicity rate, grid cells with a background seismicity rate higher than ${thr\_PD}_{\mu }$ are alarmed, as shown in red in Figure 6b. When considering both the *b* value and background seismicity rate, grid cells that are alarmed in both Figure 6a,b are marked for alarm, as shown in Figure 6c. Notably, regions such as Dali, Lijiang, Baoshan, Puer, and Yuxi exhibit higher earthquake potential.

Based on the maximum *PD*, we first identify potential high-earthquake-risk areas by focusing on regions with low *b* values, as shown in Figure 6a. Subsequently, the high-risk zones are refined by excluding areas with low background seismicity rates, as shown in Figure 6c. The retrospective analysis of two decades of data (2005–2024) shows that this method reduces the alarming rate and increases *PG* by 40% compared to forecasts based solely on *b* value, indicating improved forecasting performance by integrating background seismicity rate. Future research will focus on effectively incorporating multiple data sources, as improving observation accuracy and increasing data availability enhanced earthquake forecasting potential. One potential solution is to employ machine learning techniques to assist in decision-making. Compared to direct learning from raw observational data, data that have been effectively processed and preliminarily tested, such as the *b* value and the background seismicity rate adopted in this study, contain more useful information and may potentially lead to better forecasting outcomes.

The proposed approach involves the formulation of a comprehensive five-year earthquake prediction model that is fundamentally based on patterns of seismic activity. However, accurately determining the timing of earthquake occurrences requires additional validation through short-term prediction techniques. These techniques include the analysis of foreshocks, geophysical anomalies that may signal changes in the Earth’s crust, and other recognized earthquake precursors [48,49,50,51,52]. The feasibility of this validation process has been improved due to recent advancements in observational technologies, which allow for more precise monitoring and analysis of seismic phenomena. Moreover, middle–long-term forecasting can provide crucial insights that aid in the systematic organization of high-quality observations in potential fault zones, thus helping to enhance the overall understanding of seismic behavior. In addition, the prediction results can be helpful for regional disaster preparedness and risk management.

## 6. Conclusions

Using the earthquake catalog from the China Earthquake Networks Center, this study validates the practicality and efficacy of earthquake forecasting based on the *b* value, as proposed in the work of Wang et al. (2021) [23]. The predictive performance of both the background seismicity rate and the *b* value is comprehensively evaluated, demonstrating their effectiveness for 5-year forecasting in Yunnan Province, China. The performance of the *b* value shows a positive correlation with the predicted earthquake magnitude. The synergistic effect of combining these two predictors is also explored. By employing the threshold corresponding to the maximum *PD*, we identify high-earthquake-risk areas based on low *b* values and refine them by excluding regions with low background seismicity rates. A retrospective analysis shows this strategy can reduce the alarming rate and increase *PG* by 40% compared to the *b*-value-only forecast. A forward prediction for the period from January 2025 to December 2029 is finally derived, identifying regions with high earthquake potential, including Dali, Lijiang, Baoshan, Puer, and Yuxi.

## Figures and Tables

**Table 1:** List of earthquakes with M ≥ 5.0 in Yunnan Province during 2020–2024.

Serial Number	Time ^1^	Location			Magnitude
		Longitude	Latitude	Depth (Km)	
1	2020-05-18 21:48:00	103.17	27.20	8	5.0
2	2021-05-21 21:21:25	99.92	25.65	10	5.6
3	2021-05-21 21:48:35	99.88	25.70	10	6.5
4	2021-05-21 21:55:29	99.89	25.67	10	5.0
5	2021-05-21 22:31:10	99.97	25.62	8	5.2
6	2021-06-10 19:46:07	101.92	24.35	8	5.1
7	2021-06-12 18:00:47	97.88	24.97	16	5.0
8	2022-01-02 15:02:07	100.65	27.79	10	5.5
9	2022-11-19 01:27:34	102.26	23.37	8	5.4
10	2023-05-02 23:27:22	99.28	25.53	10	5.2

^1^ Time is given by the date (YYYY-MM-DD) and time (hh:mm:ss).

**Table 2:** Maximum *PG* and *PD* marked in Figure 5, their location, alarming rate, and detecting rate.

Magnitude	Type	Maximum Value	Alarming Rate (*μ*)	Alarming Rate (b)	Comprehensive Alarming Rate	Comprehensive Detecting Rate
M≥5.5	*PG*	26.68	0.03	0.09	0.0052	0.14
	*PD*	0.46	0.64	0.54	0.36	0.83
M≥5.0	*PG*	16.81	0.02	0.01	0.0014	0.023
	*PD*	0.38	0.48	0.89	0.43	0.81

**Table 3:** The threshold determined by maximum *PD* of M ≥ 5.5 in Figure 3c and its alarming rate, detecting rate, and *PG*.

	Threshold	Alarming Rate	Detecting Rate	*PG*	Comprehensive Alarming Rate	Comprehensive Detecting Rate	Comprehensive *PG*
*b*	0.9056	0.22	0.58	2.64	0.13	0.48	3.69
μ	0.0033	0.47	0.90	1.91			

## Data Availability

The catalog in this study was provided by the Earthquake Administration of Yunnan Province, China.
